# AI-powered hierarchical classification of ampullary neoplasms: a deep learning approach using white-light and narrow-band imaging

**DOI:** 10.1007/s00464-025-12534-2

**Published:** 2026-01-14

**Authors:** Dan Yoon, Sung Hoon Chang, Woo Hyun Paik, Chang Hyun Kim, Byeong Soo Kim, Young Gyun Kim, Hyunsoo Chung, Ji Kon Ryu, Sang Hyub Lee, In Rae Cho, Seong Ji Choi, Joo Seong Kim, Sungwan Kim, Jin Ho Choi

**Affiliations:** 1 Interdisciplinary Program in Bioengineering, Seoul National University Graduate School, Seoul, 08826 Republic of Korea; 2 Department of Internal Medicine and Liver Research Institute, Seoul National University College of Medicine, Seoul National University Hospital, Seoul, 03080 Republic of Korea; 3 Korean Society of Gastrointestinal Endoscopy Artificial Intelligence Research Group (KSGE AI Research Group), Seoul, 04050 Republic of Korea; 4 Institute of Medical and Biological Engineering, Medical Research Center, Seoul National University, Seoul, 03080 Republic of Korea; 5 Division of Gastroenterology and Hepatology, Department of Internal Medicine, Korea University College of Medicine, Seoul, 02841 Republic of Korea; 6 Division of Gastroenterology, Department of Internal Medicine, Dongguk University Ilsan Hospital, Dongguk University School of Medicine, Goyang, 10326 Republic of Korea; 7 Department of Biomedical Engineering, Seoul National University College of Medicine, Seoul, 03080 Republic of Korea

**Keywords:** Ampulla of Vater neoplasm, Endoscopic images, Narrow-band imaging, Hierarchical classification, Deep learning

## Abstract

**Background:**

Endoscopic diagnosis of Ampulla of Vater (AoV) lesions remains challenging owing to complex morphology and limited representative images, particularly for high-risk dysplastic lesions. This study aimed to develop a hierarchical deep learning framework for the stepwise classification of ampullary lesions using white-light (WL) and narrow-band endoscopic images (NBI).

**Methods:**

The framework employs three sequential binary classifications: (1) normal vs. abnormal, (2) adenoma vs. cancer, and (3) high-grade dysplasia (HGD) vs. low-grade dysplasia (LGD) within adenomas. Each stage uses EfficientNet-B4 classifiers trained independently on WL and NBI. Predictions are integrated using confidence-based voting. To overcome data scarcity and class imbalance, for HGD and cancer, we used StyleGAN2-ADA to generate synthetic images. The hierarchical model was developed using 4244 endoscopic images from 464 patients collected at Seoul National University Hospital (2693/833/718 for train/validation/test).

**Results:**

The hierarchical model achieved stage-specific accuracies of 95.6% (normal vs. abnormal), 94.4% (adenoma vs. cancer), and 92.7% (LGD vs. HGD), resulting in overall diagnostic accuracy of 92.2%. The model demonstrated excellent sensitivity of 83.3% for HGD and 87.5% for cancer, with specificities exceeding 98%. The confidence-based dual-modality approach (AUROC: 0.921) significantly outperformed single-modality approaches using WL alone (AUROC: 0.866) or NBI alone (AUROC: 0.895), by integrating their complementary diagnostic strengths. Generative adversarial network-based augmentation substantially improved sensitivity for cancer (from 87.5% to 91.7%) and HGD (from 83.3% to 86.5%), while overall accuracy increased from 94.5% to 95.1%.

**Conclusions:**

A hierarchical deep learning approach integrating dual-modality imaging and synthetic data augmentation significantly improves diagnostic performance for ampullary lesions.

**Graphical abstract:**

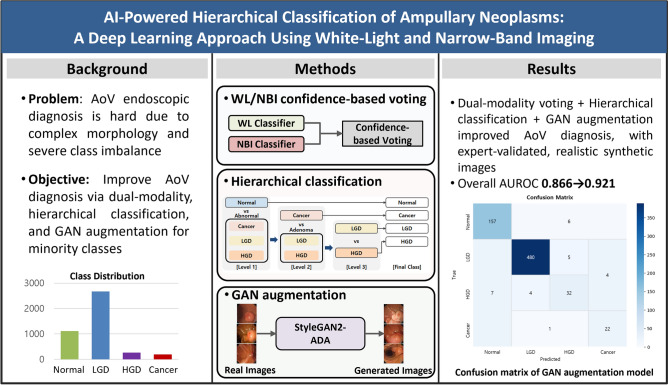

**Supplementary Information:**

The online version contains supplementary material available at 10.1007/s00464-025-12534-2.

Neoplasms of the Ampulla of Vater (AoV) are rare tumors that are currently being diagnosed with increasing frequency owing to advances in imaging techniques and expanded use of diagnostic endoscopy for healthcare screening [1]. AoV adenomas represent precancerous lesions that progress through the adenoma–carcinoma sequence [2–5]. Accurate classification into low-grade dysplasia (LGD), high-grade dysplasia (HGD), or carcinoma is essential for optimal clinical decision-making: small LGD adenomas can be managed conservatively or with endoscopic papillectomy (EP), whereas HGD and carcinoma require pancreaticoduodenectomy [6–9]. Although both HGD and carcinoma are typically managed surgically, distinguishing them remains clinically important for postoperative management and prognostication. However, this diagnostic process remains challenging given the subtle morphological features, inter-observer variability, and inconsistent image quality [10–13]. Current diagnosis relies on endoscopic tissue biopsy, which fails to represent the entire lesion with risks of mistargeting; in addition, the procedure carries potential complications including bleeding and pancreatitis [14–17]. Furthermore, the anatomically protruding structure of the AoV renders evaluation challenging with standard forward-viewing endoscopes. Despite the use of specialized endoscopes, optical diagnosis often remains extremely difficult, suggesting the need for ancillary diagnostic tools [13, 18]. Concordantly, prior work has reported wide variability in the diagnostic accuracy for AoV tumors (sensitivity 20–91%; specificity 75–83%) [19], underscoring the need for adjunctive decision-support systems.

In recent years, deep learning-based artificial intelligence (AI) has shown promise for automating medical image analysis [20–25]. While substantial research has focused on the optical diagnosis of gastrointestinal polyps with white-light (WL) endoscopy [24, 25], the application of AI for the evaluation of AoV tumors remains limited. Narrow-band imaging (NBI), which visualizes pit patterns on the mucosal surface, enables a more detailed prediction of polyp characteristics and assessment of dysplasia grade [26, 27]. Despite the proven utility of NBI for other gastrointestinal lesions [22, 28], its application in AI-assisted diagnosis of AoV tumors remains underexplored. This highlights a significant clinical need, as AoV tumors present unique diagnostic challenges given their relatively low frequency and poor visualization via forward-viewing endoscopy, requiring substantial experience and time for physicians to achieve diagnostic proficiency [13, 18, 29]. Hence, AI model assistance could provide substantial support for clinical decision-making in real-world practice [30, 31].

This study aimed to develop a novel AI-powered optical endoscopic diagnosis model for AoV neoplasms using both WL and NBI images. Our approach employs a hierarchical three-step classification system designed to mirror the clinical diagnostic workflow. To address the challenges of limited data and class imbalance inherent in rare tumors, we implemented a confidence-based ensemble method and synthetic data augmentation techniques.

## Methods

### Dataset and preprocessing

We collected a dataset of endoscopic images from patients diagnosed with ampullary lesions at Seoul National University Hospital between January 2010 and May 2025. Each image was labeled according to the corresponding histopathological diagnosis as normal (or non-neoplastic), adenoma with low-grade dysplasia (LGD), adenoma with high-grade dysplasia (HGD), or carcinoma. The dataset also included retrospectively acquired data, obtained under institutional IRB approval with a consent waiver (IRB No. H-2406-011-1540). Evaluations of AoV lesions were performed by five experienced endoscopists using standard side-viewing duodenoscopes (TJF-260 and JF-260; Olympus Optical Co. Ltd., Tokyo, Japan). Endoscopic images were captured using video endoscopy systems (EVIS LUCERA CLV 260; Olympus Medical Systems Co. Ltd., Tokyo, Japan). Histology served as the diagnostic standard, and pathology from EP resection was prioritized over pre-EP biopsy results.

To ensure robust model development and evaluation, we randomly divided the dataset at the patient-level into training, validation, and test sets in a 6:2:2 ratio, ensuring no patient overlap across subsets. WL and NBI images were acquired from the same lesions within the same endoscopic session when available; however, frame counts per modality varied and some lesions did not have NBI, so the modality sets are lesion matched but not strictly one to one paired. The same patient-level split was applied to both WL and NBI, maintaining identical patient lists across modalities. Because patients contributed different numbers of images, the 6:2:2 target was approximated while balancing class distribution. This allocation ensured adequate per-class sample sizes, including minority HGD and cancer, in the held-out test set while preserving WL/NBI class balance. In total, we used 2,693/833/718 images for training, validation, and testing; detailed class- and modality-wise counts are summarized in Supplementary Table S1. Images were cropped to include only the endoscopic view, excluding any identifiable patient information. Data augmentation techniques, including random cropping, horizontal flipping, and color jittering, were applied during training to enhance the generalizability of the models [32].

### Hierarchical classification with confidence-based voting

**Fig. 1:**
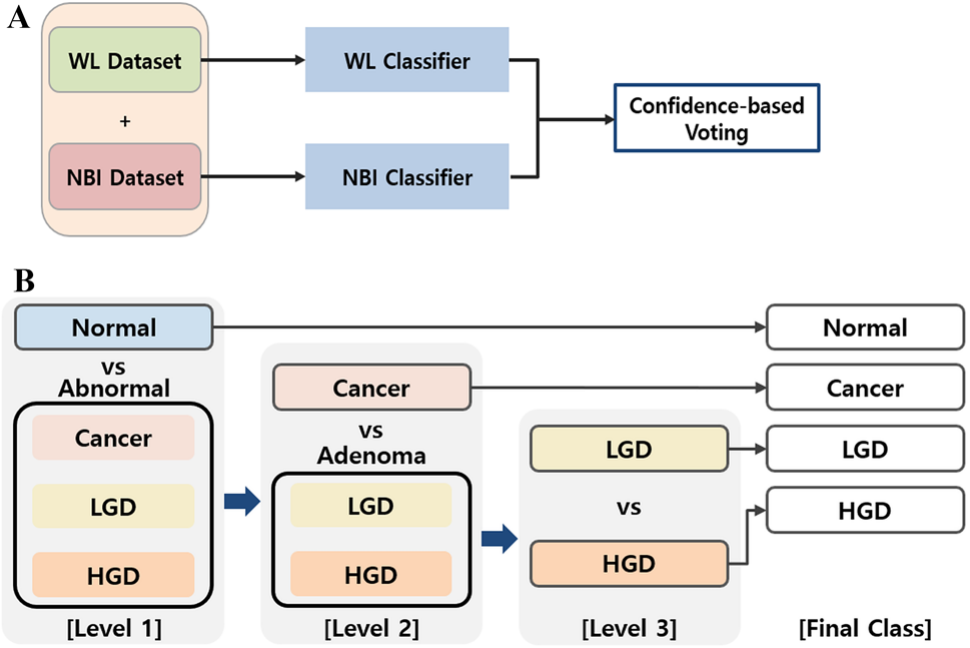
Overview of the hierarchical classification pipeline. **A** Confidence-based voting with white-light (WL) classifier and narrow-band imaging (NBI) classifier. **B** Hierarchical classification steps for ampullary lesion diagnosis; level 1: normal vs abnormal (including cancer, low-grade dysplasia (LGD), and high-grade dysplasia (HGD)), level 2: Cancer vs adenoma (including LGD and HGD), level 3: LGD vs HGD

To align with the clinical decision-making process, we implemented a three-stage hierarchical classification strategy using three binary classification steps: (1) normal vs. abnormal, (2) adenoma vs. cancer (among abnormal cases), and (3) LGD vs. HGD (among adenomas). This architecture reflects clinical diagnostic triage and reduces decision complexity relative to a single multi-class model, which is advantageous under severe class imbalance. Each binary task was handled by independently trained classifiers with confidence-based voting (Fig. 1A) [33, 34]. The final diagnosis for each case was inferred through sequential evaluation of the three stages, yielding one of the following final classes: normal, LGD, HGD, or cancer (Fig. 1B).

**Fig. 2:**
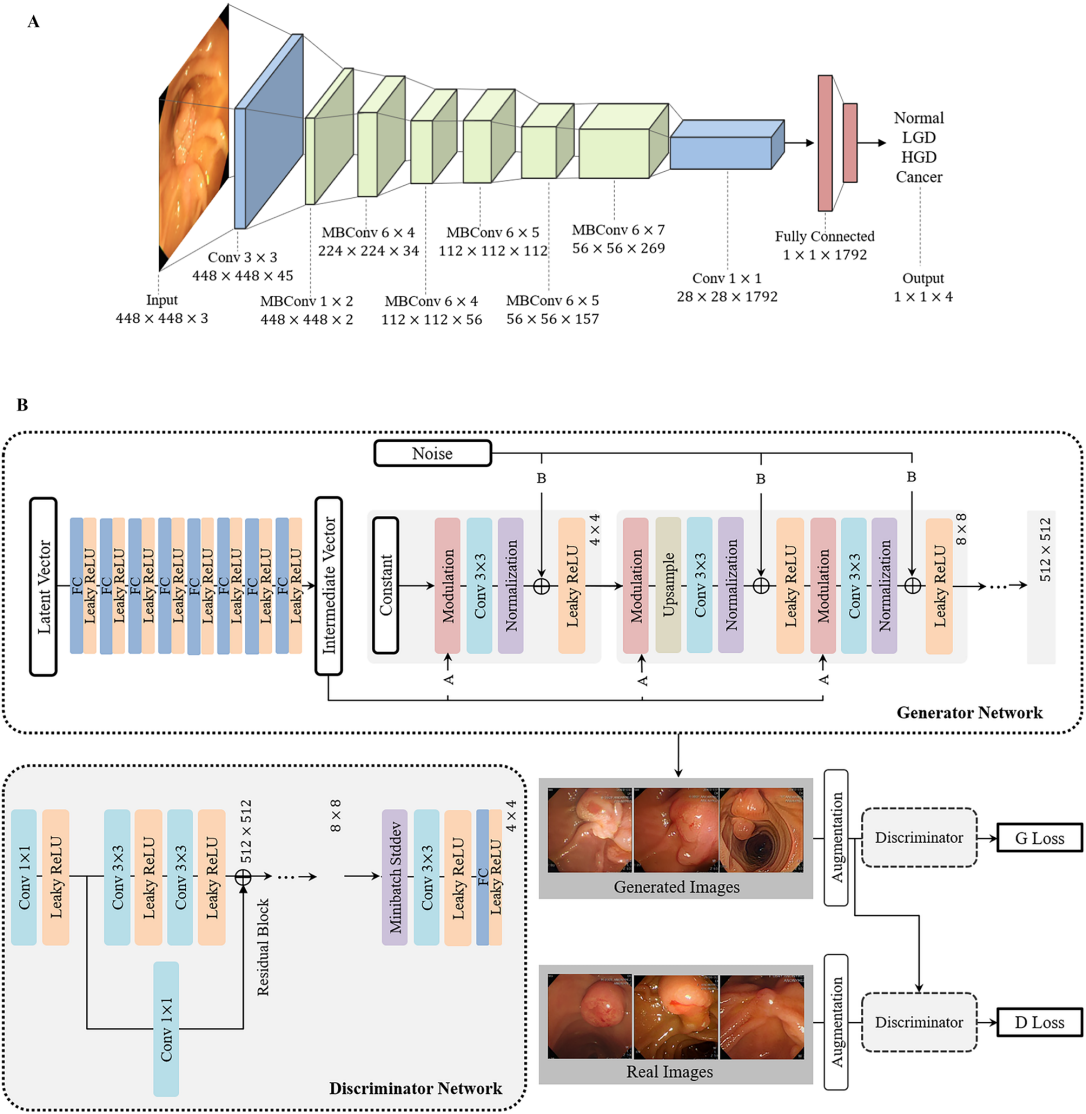
Model architectures used for AoV lesion classification and GAN-based image synthesis. **A** EfficientNet-B4 architecture used for classifying Ampulla of Vater (AoV) lesions, employing hierarchical classification tasks to distinguish between normal vs. abnormal, adenoma vs. cancer, and high-grade vs. low-grade dysplasia. **B** StyleGAN2-ADA architecture for synthesizing high-quality endoscopic images, utilizing adaptive discriminator augmentation to improve the generalizability of the model with limited data

To enhance diagnostic precision, particularly for high-risk lesions, we incorporated information from both WL and NBI modalities. For each classification stage, two separate EfficientNet-B4 models [35] were trained independently on WL and NBI images (Fig. 2A). During inference, the final prediction was determined by a confidence-based voting mechanism, selecting the class with the higher confidence score from the two modality-specific models. Detailed architectural and training specifications of EfficientNet-B4 are provided in the supplementary materials. All training was performed at the frame level, ensuring no patient overlap across the training, validation, and test sets.

### Data augmentation using GAN

To address class imbalance and limited sample size for HGD and cancer classes, which have naturally limited distribution, we generated synthetic endoscopic images using a StyleGAN2-ADA model (Fig. 2B). StyleGAN2-ADA was trained using the original image distribution of underrepresented classes (particularly HGD and cancer) to create high-resolution (512 × 512) synthetic samples for data augmentation [36, 37]. To prevent leakage, the GAN was trained exclusively on the patient-level training split, and we used the WL modality only, as each minority class had more than 100 WL training images available. Classification dataset with the same training, validation, and test set was used to train StyleGAN2-ADA. To generate consistent lesion images, noisy images containing surgical tools or post-EP changes were excluded from the GAN training dataset. Detailed GAN training parameters, including network configuration, optimizer settings, and loss functions, are described in the supplementary materials.

### Evaluation metrics

Evaluation metrics included accuracy, precision, recall, and F1-score, calculated separately for each classification stage. In addition, we computed confusion matrices and hierarchical classification consistency across stages. All evaluations were conducted at the frame level, and there was no patient overlap among the training, validation, and test sets.

To quantitatively evaluate the quality of the generated images, we measured Fréchet inception distance (FID) [38], which quantifies the distributional similarity between real and synthetic images, and signal-to-noise ratio (SNR) [39], which measures image clarity. Detailed information is described in the supplementary materials.

## Results

### Baseline characteristics of study patients

This study analyzed 4244 endoscopic images from 464 patients with histopathologically confirmed diagnoses, collected at Seoul National University Hospital between January 2010 and May 2025 (Table 1). Among the study patients, there were slightly more patients over 65 years (254, 54.7%) than those 65 years or younger (210, 45.3%). Males comprised 52.8% of the cohort (245 patients), with females accounting for the remainder (219, 47.2%). Most AoV abnormalities were initially detected through direct visualization of abnormal morphology during health check-up gastroscopy (306 cases, 65.9%). Abdominal imaging findings prompted further investigation in 130 patients (28.0%), while clinical symptoms led to diagnosis less frequently. The majority of lesions were small, with 374 (80.6%) lesions measuring 2 cm or less in diameter. Histopathological examination revealed considerable variation in tissue characteristics. Normal or non-neoplastic tissue was found in 172 patients (37.1%), while LGD represented the most frequent pathological finding (209 patients, 45.0%). More advanced changes were less common, with HGD present in 36 patients (7.8%) and invasive cancer in 47 patients (10.1%). Eleven patients (2.4%) carried a diagnosis of familial adenomatous polyposis.

**Table 1:** Characteristics of AoV lesion dataset

Characteristics	*N* (%)
Total number of patients	464 (100)
Age	
> 65 years	254 (54.7)
≤ 65 years	210 (45.3)
Sex	
Male	245 (52.8)
Female	219 (47.2)
AoV abnormality detection	
Abnormal morphology of AoV at gastroscope	306 (65.9)
Abdominal imaging abnormality	130 (28.0)
Abdominal pain	11 (2.4)
Suspicious jaundice	6 (1.3)
Dyspepsia	6 (1.3)
Others	4 (0.9)
AoV mass size	
> 2 cm	89 (19.2)
≤ 2 cm	374 (80.6)
AoV mass histology (endoscopic biopsy)	
Normal, Non-neoplastic lesion	172 (37.1)
LGD	209 (45.0)
HGD	36 (7.8)
Cancer	47 (10.1)
Familial adenomatous polyposis	11 (2.4)

### Classification performance of WL, NBI, and confidence-based voting models

**Fig. 3:**
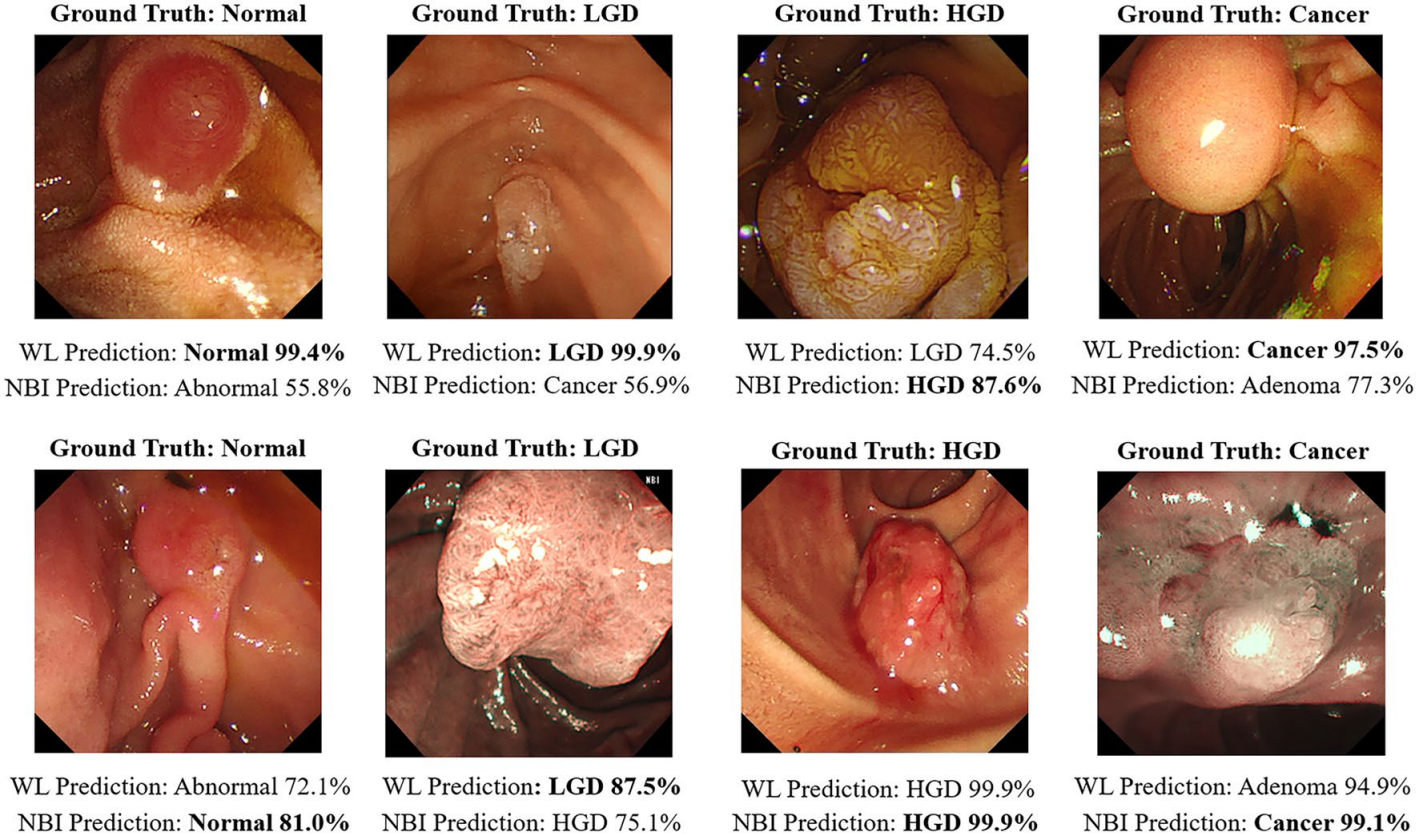
Predictions made by WL and NBI models for each lesion image, along with confidence scores. For each image, predictions and confidence scores from both the WL and NBI models are shown. The final decision used for the classification is highlighted in bold, based on the confidence-based voting strategy

Confidence-based voting, which integrates predictions from both WL and NBI models, significantly improved diagnostic performance compared to using either modality alone. On the WL test set (*n* = 547 images), the voting model achieved a higher accuracy and area under the receiver operating characteristic curve (AUROC) (91.8% and 0.9136, respectively) compared to the WL-only model (87.9% and 0.8662). Similarly, on the NBI test set (*n* = 171 images), the voting model (accuracy: 93.6%, AUROC: 0.9208) demonstrated superior performance over the NBI-only model (88.9%, 0.8948) (Supplementary Table S2). This improvement stems from leveraging the complementary strengths of each modality; the WL model generally performed better in detecting normal and LGD lesions, while the NBI model showed superior accuracy for HGD and cancer. Specifically, for the LGD lesion shown in the NBI image, the WL model provided a higher confidence score and correctly classified the lesion as LGD (87.5%), prevented a potential misdiagnosis from incorrect HGD prediction (75.1%) of the NBI model (Fig. 3). Conversely, for the HGD lesion shown in the WL image, the NBI model yielded a more confident and accurate prediction than the WL model (HGD 87.6% vs. LGD 74.5%). Based on these results, dual-modality confidence-based voting demonstrated superior performance by minimizing diagnostic errors through cross-validation between modalities. This integration of modality-specific features enhanced clinical utility and diagnostic precision.

**Fig. 4:**
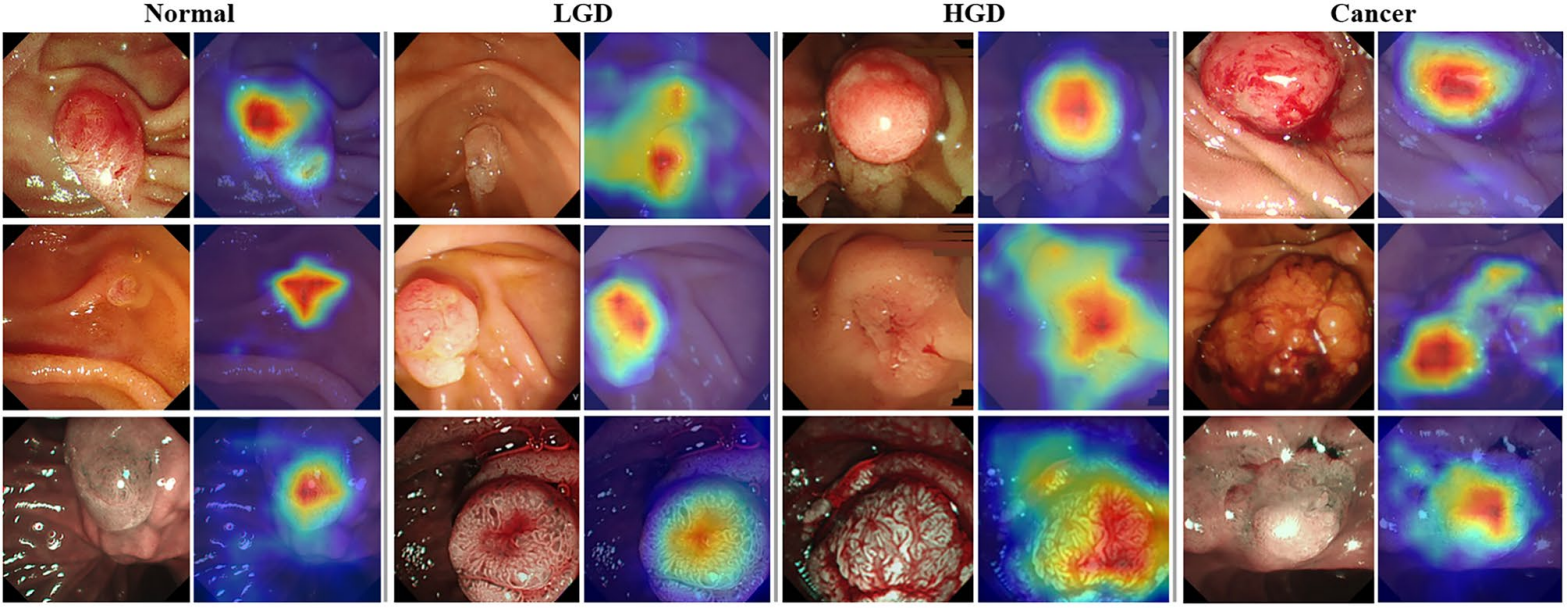
Grad-CAM visualizations for the confidence-based voting model across different lesion types. Top two rows: white-light (WL) examples. Bottom row: narrow-band imaging (NBI) examples. Heatmaps illustrate lesion-focused attention for representative cases across normal, LGD, HGD, and cancer

We employed Grad-CAM to visualize the discriminative regions that contributed to each prediction [40]. The heatmaps (Fig. 4**)** show that the models focused on clinically relevant lesion areas during classification, indicating reliable attention to diagnostically important regions.

### Hierarchical classification performance of AoV lesions

**Fig. 5:**
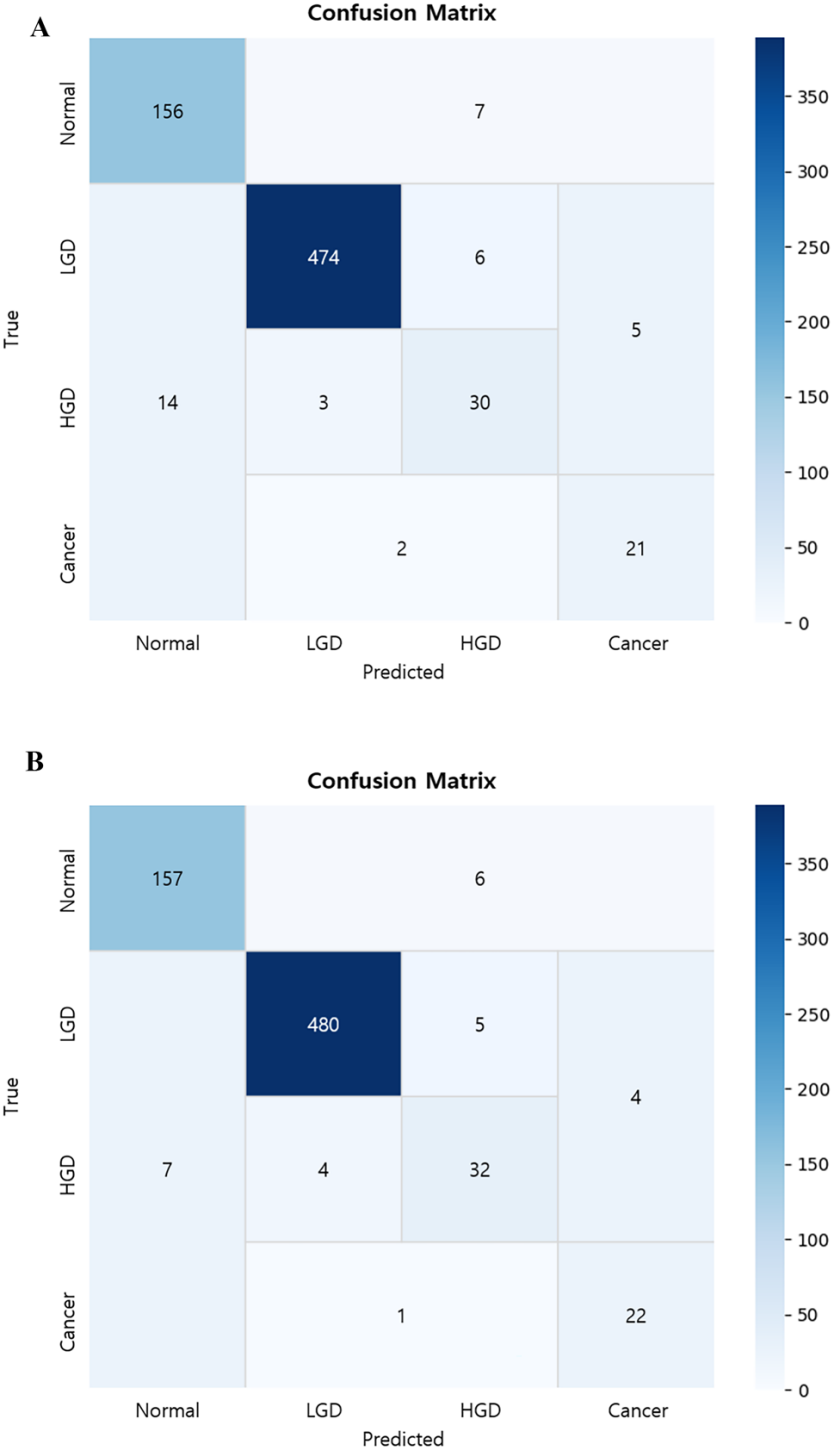
Confusion matrices for the hierarchical classification of AoV lesions. **A** Results obtained using the original dataset without augmentation, and **B** results obtained using the augmented dataset with synthetic images generated via StyleGAN2-ADA

The proposed hierarchical classification system exhibited high performance across all three classification stages. In the first stage (normal vs. abnormal), the confidence-based voting classifier achieved an accuracy of 95.6% on the WL test set and 95.9% on the NBI test set. In the second stage (adenoma vs. cancer), the model yielded accuracies of 94.0% and 96.2% for the WL and NBI test sets, respectively. For the third stage (LGD vs. HGD), the model achieved an accuracy of 93.0% on the WL test set and 91.4% on the NBI test set. The resulting overall diagnostic accuracy, defined as the accuracy of the final class assignment, was 91.8% for the WL test set and 93.6% for the NBI test set (Table 2). Figure 5A presents the detailed confusion matrix for each class. Compared with a conventional multi-class classification model that directly assigns one of the four classes in a single step, the proposed hierarchical approach yielded superior performance across all the evaluation metrics (Table 3). The stepwise structure enabled finer discrimination at each diagnostic decision point, contributing to improved overall accuracy and robustness, particularly in distinguishing clinically challenging categories such as HGD and cancer.

**Table 2:** Classification performance of AoV lesion diagnosis using white-light (WL), narrow-band imaging (NBI), and confidence-based voting models (*n* = number of images)

WL test set (*n* = 547)										
	WL Model (95% CI)					Confidence-based voting model (95% CI)				
	Sensitivity	Specificity	PPV	NPV	F1-score	Sensitivity	Specificity	PPV	NPV	F1-score
Normal (*n* = 98)	0.8776 [0.8127, 0.9276]	0.9421 [0.9170, 0.9611]	0.7679 [0.7025, 0.8333]	0.9816 [0.9693, 0.9939]	0.8191 [0.7709, 0.8612]	0.8878 [0.8210, 0.9332]	0.9621 [0.9415, 0.9777]	0.8365 [0.7770, 0.8961]	0.9842 [0.9729, 0.9955]	0.8614 [0.8181, 0.8993]
LGD (*n* = 397)	0.9068 [0.8735, 0.9327]	0.8933 [0.8445, 0.9313]	0.9574 [0.9372, 0.9776]	0.7836 [0.7322, 0.8350]	0.9314 [0.9114, 0.9449]	0.9496 [0.9248, 0.9696]	0.8933 [0.8519, 0.9346]	0.9563 [0.9364, 0.9763]	0.8701 [0.8260, 0.9142]	0.9529 [0.9247, 0.9663]
HGD (*n* = 29)	0.5862 [0.4089, 0.7296]	0.9846 [0.9708, 0.9929]	0.6800 [0.5243, 0.8357]	0.9847 [0.9747, 0.9947]	0.6296 [0.5195, 0.7774]	0.6552 [0.4832, 0.7937]	0.9884 [0.9796, 0.9971]	0.7600 [0.6175, 0.9024]	0.9808 [0.9697, 0.9920]	0.7037 [0.5677, 0.8319]
Cancer (*n* = 23)	0.6667 [0.4903, 0.8144]	0.9769 [0.9602, 0.9869]	0.6000 [0.4482, 0.7518]	0.9826 [0.9720, 0.9932]	0.6316 [0.5245, 0.7756]	0.7037 [0.5189, 0.8365]	0.9885 [0.9799, 0.9972]	0.7600 [0.6152, 0.9048]	0.9847 [0.9746, 0.9947]	0.7308 [0.5984, 0.8421]

NBI test set (*n* = 171)										
	NBI Model (95% CI)					Confidence-based voting model (95% CI)				
	Sensitivity	Specificity	PPV	NPV	F1-score	Sensitivity	Specificity	PPV	NPV	F1-score
Normal (*n* = 65)	0.8769 [0.7943, 0.9421]	0.9245 [0.8651, 0.9632]	0.8769 [0.8071, 0.9467]	0.9245 [0.8794, 0.9695]	0.8769 [0.8248, 0.9207]	0.9692 [0.9003, 0.9927]	0.9528 [0.9037, 0.9831]	0.9692 [0.9325, 0.9947]	0.9806 [0.9567, 0.9920]	0.9692 [0.9302, 0.9874]
LGD (*n* = 98)	0.8876 [0.8296, 0.9495]	0.8904 [0.7994, 0.9331]	0.8878 [0.8296, 0.9460]	0.8553 [0.7890, 0.9216]	0.8877 [0.8368, 0.9307]	0.9286 [0.8653, 0.9689]	0.9589 [0.9044, 0.9895]	0.9192 [0.8692, 0.9693]	0.8961 [0.8364, 0.9528]	0.9239 [0.8928, 0.9537]
HGD (*n* = 7)	0.7143 [0.3491, 0.9681]	0.9756 [0.9451, 0.9922]	0.6250 [0.3112, 0.9388]	0.9877 [0.9727, 1.000]	0.6667 [0.4776, 0.8572]	0.7143 [0.4013, 1.0000]	0.9817 [0.9517, 0.9948]	0.7143 [0.4013, 0.9852]	0.9877 [0.9727, 0.9886]	0.7143 [0.4628, 0.9678]
Cancer (*n* = 1)	1.0000 [0.7151, 1.0000]	0.9941 [0.9733, 0.9999]	0.5000 [0.2911, 0.7089]	1.0000 [0.7168, 1.0000]	0.6667 [0.4546, 0.8784]	1.0000 [0.7225, 1.0000]	1.0000 [0.9823, 1.0000]	1.0000 [0.7228, 1.0000]	1.0000 [0.9789, 1.0000]	1.0000 [0.7226, 1.0000]

**Table 3:** Comparison of classification performance between multi-class and hierarchical models for AoV lesion diagnosis (*n* = number of images)

	Multi-class model (95% CI)					Hierarchical model (95% CI)				
	Sensitivity	Specificity	PPV	NPV	F1-score	Sensitivity	Specificity	PPV	NPV	F1-score
Normal (*n* = 163)	0.9202 [0.8848, 0.9556]	0.9010 [0.8774, 0.9246]	0.8876 [0.8471, 0.9281]	0.9728 [0.9594, 0.9862]	0.9036 [0.8728, 0.9433]	0.9570 [0.9304, 0.9835]	0.9875 [0.9787, 0.9963]	0.9176 [0.8824, 0.9528]	0.9892 [0.9810, 0.9974]	0.9369 [0.9178, 0.9563]
LGD (*n* = 495)	0.9455 [0.9260, 0.9650]	0.8924 [0.8584, 0.9264]	0.9512 [0.9326, 0.9698]	0.8805 [0.8452, 0.9158]	0.9484 [0.9329, 0.9627]	0.9576 [0.9402, 0.9750]	0.9552 [0.9325, 0.9779]	0.9834 [0.9722, 0.9946]	0.9342 [0.9073, 0.9611]	0.9703 [0.9578, 0.9799]
HGD (*n* = 36)	0.6667 [0.5333, 0.8001]	0.9912 [0.9846, 0.9978]	0.7742 [0.6469, 0.9015]	0.9825 [0.9734, 0.9916]	0.7164 [0.6022, 0.8119]	0.8333 [0.7279, 0.9387]	0.9927 [0.9868, 0.9986]	0.7894 [0.6771, 0.9017]	0.9941 [0.9888, 0.9994]	0.8108 [0.7240, 0.8814]
Cancer (*n* = 24)	0.7500 [0.6262, 0.8738]	0.9870 [0.9792, 0.9947]	0.6667 [0.5396, 0.7938]	0.9913 [0.9848, 0.9978]	0.7059 [0.6038, 0.8002]	0.8750 [0.7804, 0.9696]	0.9928 [0.9869, 0.9987]	0.7500 [0.6354, 0.8646]	0.9971 [0.9933, 1.0000]	0.8077 [0.7217, 0.8689]

We evaluated the impact of data augmentation using StyleGAN2-ADA-generated synthetic images for the underrepresented HGD and cancer classes. The overall accuracy increased from 94.5% to 95.1%, and notably, the recall rates for HGD and cancer improved from 83.3% to 86.5% and from 87.5% to 91.7%, respectively (Table 4**)**. Performance improvement was particularly significant in the third stage (LGD vs. HGD), where limited data had previously constrained model sensitivity. The use of augmented training data consistently enhanced the classification accuracy **(**Fig. 5B).

**Table 4:** Hierarchical classification performance of AoV lesion diagnosis with confidence-based voting model trained on the original and GAN-augmented datasets (*n* = number of images)

	Original dataset (95% CI)			Augmented dataset (95% CI)		
	Recall	Precision	F1-score	Recall	Precision	F1-score
Normal (*n* = 163)	0.9570 [0.9304, 0.9835]	0.9176 [0.8824, 0.9528]	0.9369 [0.9178, 0.9563]	0.9632 [0.9386, 0.9878]	0.9573 [0.9310, 0.9836]	0.9602 [0.9404, 0.9774]
LGD (*n* = 495)	0.9576 [0.9402, 0.9750]	0.9834 [0.9722, 0.9946]	0.9703 [0.9578, 0.9799]	0.9697 [0.9549, 0.9845]	0.9798 [0.9676, 0.9920]	0.9747 [0.9646, 0.9840]
HGD (*n* = 36)	0.8333 [0.7279, 0.9387]	0.7894 [0.6771, 0.9017]	0.8108 [0.7240, 0.8814]	0.8611 [0.7481, 0.9742]	0.8649 [0.7682, 0.9616]	0.8750 [0.7955, 0.9381]
Cancer (*n* = 24)	0.8750 [0.7804, 0.9696]	0.7500 [0.6354, 0.8646]	0.8077 [0.7217, 0.8689]	0.9167 [0.8377, 0.9957]	0.8148 [0.7101, 0.9195]	0.8628 [0.7889, 0.9367]

However, the hierarchical nature of the classification framework implies that errors made in the earlier stages propagate through the pipeline. For example, if a lesion is misclassified as normal in the first stage (normal vs. abnormal), it is excluded from subsequent stages, preventing the possibility of correcting the diagnosis in the later steps. This sequential dependency underscores the critical importance of maintaining high sensitivity in the first classification stage, even if the model could have correctly classified the lesion in the later stages.

### Synthetic endoscopic image augmentation using StyleGAN2-ADA

All synthetic HGD and AoV cancer images generated by the StyleGAN2-ADA were independently reviewed by three clinical experts and confirmed to be morphologically plausible prior to inclusion in the training set (Supplementary Figure S1). The validated images successfully preserved key lesion features, such as mucosal irregularity, elevation, and discoloration. Despite the limited number of original HGD (*n* = 117) and cancer (*n* = 166) samples, the GAN-generated images successfully maintained visual consistency with real-world endoscopic findings. While the diversity of lesion appearances in the synthetic dataset was limited by the inherent variability of the training data, the addition of these images significantly improved the classification performance, particularly for rare and clinically significant lesions. Furthermore, a quantitative assessment confirmed the high fidelity of synthetic images, which maintained the SNR comparable to that of the real images (Supplementary Figure S2). FID score improved from 459.82 at initialization to 61.20 at convergence, indicating substantially closer alignment to the real image distribution. For each of the HGD and cancer classes, we generated 2,000 WL candidate images and retained 1000 per class after independent expert review; only these approved images were added to the WL training set. GANs were trained exclusively on real WL images from the patient-level training split to prevent any information leakage, and both the validation and test sets included real images only. Detailed computed results can be found in the supplementary materials.

## Discussion

We developed a hierarchical classification framework for diagnosing ampullary lesions from endoscopic images, incorporating confidence-based voting and GAN-based data augmentation strategies. Our approach structures the diagnostic process into three clinically meaningful binary classification tasks, aligning the AI pipeline with real-world clinical workflows and enhancing its potential for clinical application.

The main contributions of this work focus on two key innovations. First, we incorporated a dual-modality inference mechanism with a confidence-based voting strategy that leverages the complementary strengths of WL and NBI. Compared with a previous study that relied solely on WL images, our approach demonstrates the importance of including NBI data [23]. Previous studies also reported improvement in differential diagnosis for gastrointestinal polyps with better accuracy using WL and NBI [41–43]. Models trained on WL images alone often show relatively low sensitivity for more advanced lesions such as HGD and cancer [44]. Our confidence-based voting method directly reflects how different modalities contribute to clinical decisions and improves the final diagnostic accuracy by selecting the prediction with the highest confidence across models [33, 34]. Second, we generated synthetic endoscopic images specifically tailored to the morphological characteristics of rare lesions, effectively addressing class imbalance—a persistent challenge in medical imaging datasets. In the absence of sufficient real-world data for extremely rare classes, such as AoV tumors, the incorporation of expert-validated GAN-generated samples is critical to enabling balanced learning and improving model performance, consistent with previous work conducted in similar scenarios [45, 46]. This not only improved recall for HGD (from 83.3% to 86.5%) and cancer (from 87.5% to 91.7%) but also resulted in overall accuracy gains. To ensure clinical validity and mitigate potential overfitting, all GAN-generated images were independently reviewed and validated by three board-certified endoscopy specialists before being incorporated into model training. The quality of the synthetic images was quantitatively confirmed via FID and SNR analyses. High-fidelity synthesis in a clinical setting is strengthened by broader real-world diversity; expanding multicenter data improves convergence and realism. Under limited data, we restricted generation to WL with artifact filtering and tuned hyperparameters to balance global realism against lesion-detail fidelity. We enforced train-only synthesis and capped the synthetic ratio via experiments varying the synthetic-to-real proportion, and saliency checks supported lesion-focused behavior. Overall, by integrating the two imaging modalities and addressing class imbalance with high-quality synthetic augmentation, we achieved significant improvements in diagnostic accuracy. This demonstrates the clinical utility and scalability of our approach, particularly for challenging and rare lesion types that are difficult to handle using conventional single-modality or class-imbalanced datasets. To further raise ceiling performance, we will extend augmentation to the major classes and perform controlled experiments on the number of synthetic samples per class to identify performance plateaus.

While pancreaticoduodenectomy remains the treatment of choice for ampullary cancer, its substantial morbidity warrants careful consideration, particularly when EP may be appropriate for lesions without confirmed malignancy [8, 47, 48]. Clinical guidelines support the use of endoscopic treatment in selected patients with ampullary neoplasms, highlighting the importance of accurate risk stratification [49]. However, biopsy-based decision-making is inherently limited, as it relies on partial tissue sampling and may miss focal malignancy. Our AI-driven optical biopsy system represents a feasible and effective approach to improve diagnostic accuracy at the time of endoscopy, thereby supporting more appropriate selection between EP and surgery. By enhancing the precision of risk stratification, this tool may contribute to defining the proper indications for EP and determining its optimal timing. Future research should focus on two key directions: external validation of our model through prospective multicenter studies and evaluation of its ability to predict post-EP outcomes based on endoscopic findings. These efforts will be essential for optimizing the clinical application of optical diagnosis in the management of ampullary tumors. In parallel, we plan prospective multicenter collection to broaden device and patient diversity and to increase the proportion of NBI images so that both modalities are well represented. In addition, a prospective reader study is scheduled to assess the utility of decision-support AI by contrasting unaided with AI-assisted assessments, and to directly compare its diagnostic accuracy with expert endoscopists. We will also incorporate video-based temporal context and lesion-level aggregation and apply probability calibration with uncertainty estimation, and adopt diversified ensembling to stabilize high specificity operating points.

Despite the promising results, this study has several limitations. First, the dataset was derived from a single center with a relatively small sample size, and the analysis was performed retrospectively without external validation. While our models showed high performance internally, validation on external, multicenter cohorts is necessary to assess generalizability. Second, the current framework relies solely on supervised learning using labeled image data. Exploring semi-supervised or self-supervised strategies may help reduce annotation burden and increase robustness. Third, our approach was developed using side-viewing endoscopic images specific to the AoV. As a result, direct application to forward-viewing, standard EGD scopes may be limited. Fourth, the use of GAN-based data augmentation introduces a potential risk of overfitting to synthetic patterns, underscoring the need for external validation for rare lesion types. Because synthesis was derived from the same source dataset, some risk of overfitting may remain despite our mitigation strategies. To further reduce this risk, larger multicenter dataset collection is required. Fifth, the hierarchical structure of the classification pipeline, while improving interpretability and accuracy, is inherently sensitive to errors made in the earlier stages. Misclassification in the first stage (normal vs. abnormal) prevents re-evaluation in subsequent steps, which may be problematic in clinical practice. Sixth, our model does not incorporate clinical metadata, which could be integrated in future work to potentially enhance diagnostic performance. Lastly, our method was developed using still images. While this provides an efficient benchmark for algorithm development, real-time application in clinical settings would require testing on continuous video data. Future studies should explore model deployment and performance on full endoscopic video streams to validate its utility in real-world scenarios.

## Conclusions

This study demonstrated the utility of a hierarchical classification framework that integrates modality-specific inference with confidence-based voting and synthetic data augmentation for AoV tumor diagnosis. By incorporating expert-validated synthetic images and combining predictions across WL and NBI, our approach addresses key challenges, such as data scarcity and class imbalance, thereby improving diagnostic performance. Our results support the potential role of AI-augmented endoscopy in assisting clinical decision-making and suggest broader applicability of generative augmentation techniques in medical imaging. Future research should focus on prospective validation across multicenter cohorts and explore whether optical diagnosis can aid in predicting post-EP outcomes, both of which will be important for advancing individualized management of AoV tumors.

## Supplementary Information

Below is the link to the electronic supplementary material.

Supplementary file1 (DOCX 575 KB)
