# Supplementary material for: AI-powered hierarchical classification of ampullary neoplasms: a deep learning approach using white-light and narrow-band imaging
Source: Surg Endosc. 2026 Jan 14;40(4):2902–13. doi: 10.1007/s00464-025-12534-2 (PMC13053556; doi:10.1007/s00464-025-12534-2)
Supplement: Supplementary file 1 — Supplementary file1 (DOCX 575 KB) [file 464_2025_12534_MOESM1_ESM.docx]

**Supplementary information**

**AI-Powered Hierarchical Classification of Ampullary Neoplasms: A Deep Learning Approach Using White-Light and Narrow-Band Imaging**

*Dan Yoon^1,*^, Sung Hoon Chang^2,*^, Woo Hyun Paik^2,3^, Chang Hyun Kim^2^, Byeong Soo Kim^4^, Young Gyun Kim^4^, Hyunsoo Chung^2,3^, Ji Kon Ryu^2^, Sang Hyub Lee^2^, In Rae Cho^2^, Seong Ji Choi^3,5^, Joo Seong Kim^3,6^, Sungwan Kim^1,4,7,†^, Jin Ho Choi^2,3,†^*

^1^Interdisciplinary Program in Bioengineering, Seoul National University Graduate School, Seoul, Republic of Korea

^2^Department of Internal Medicine and Liver Research Institute, Seoul National University College of Medicine, Seoul National University Hospital, Seoul, Republic of Korea

^3^Korean Society of Gastrointestinal Endoscopy Artificial Intelligence Research Group (KSGE AI Research Group)

^4^Institute of Medical and Biological Engineering, Medical Research Center, Seoul National University, Seoul, Republic of Korea

^5^Division of Gastroenterology and Hepatology, Department of Internal Medicine, Korea University College of Medicine, Seoul, Republic of Korea

^6^Division of Gastroenterology, Department of Internal Medicine, Dongguk University Ilsan Hospital, Dongguk University School of Medicine, Goyang, Republic of Korea

^7^Department of Biomedical Engineering, Seoul National University College of Medicine, Seoul, Republic of Korea

* These authors contributed equally to this work.

† These authors contributed equally to this work and are co-corresponding authors.

Co-corresponding author: Sungwan Kim

Department of Biomedical Engineering, Seoul National University College of Medicine, Seoul 03080, Republic of Korea

E-mail: [sungwan@snu.ac.kr](mailto:sungwan@snu.ac.kr)

Co-corresponding author: Jin Ho Choi

Department of Internal Medicine and Liver Research Institute, Seoul National University College of Medicine, Seoul 03080, Republic of Korea

E-mail: jinhchoi@snu.ac.kr

**EfficientNet-B4 Architecture and Training Configuration**

EfficientNet-B4, which is a convolutional neural network based on a compound scaling method, was selected for its strong balance between performance and computational efficiency [1]. Each model was initialized with ImageNet-pretrained weights and fine-tuned on our medical dataset. The final classification layer comprises a single output neuron with a sigmoid activation function. Binary cross-entropy loss was used in all the tasks. The loss function for each binary classification task is defined as [1]

$\mathcal{L}_{BCE}=-\frac{1}{N}\sum_{i=1}^{N} \left[ y_{i}\log\left( \hat{y}_{i} \right)+\left( 1-y_{i} \right)\log\left( 1-\hat{y}_{i} \right) \right] (1)$

where$y_{i}$​ is the ground truth label, and $\hat{y}_{i}$is the predicted probability for the *i*^th^ image. All the models were trained using the Adam optimizer with a learning rate of 0.0001 and early stopping based on the validation loss.

**StyleGAN2-ADA Training Configuration**

We adopted the StyleGAN2-ADA configuration with adaptive discriminator augmentation to prevent overfitting and trained the network for 100,000 iterations using the Adam optimizer ($\beta_{1}=0$*,* $\beta_{2}=0.99$) with an initial learning rate of 0.002. The generator and discriminator losses can be defined as [2]

$\mathcal{L}_{G}=-\mathbb{E}_{z}\left[ D\left( G\left( z \right) \right) \right],$ (2)

$\mathcal{L}_{D}=\mathbb{E}_{x}\left[ max(0, 1-D\left( x \right))]+\mathbb{E}_{z}[max(0, 1+D\left( G\left( z \right) \right)) \right]$ (3)

To validate morphological fidelity, two clinical experts independently reviewed the generated images. Reviewed images were retained and used to augment the training set. The number of synthetic samples for HGD and cancer was matched with that of the largest class (LGD), ensuring balanced representation across training classes.

**Fréchet Inception Distance (FID) Calculation**

To quantitatively assess the similarity between real and synthetic images, we computed the Fréchet inception distance (FID) [3], which measures the distance between the two distributions in the high-dimensional feature space of an Inceptionv3 classifier with Fréchet inception distance (FID) [3]. If the activations on the real and synthesized data are $N(m, C)$ and $N(m_{\omega},C_{\omega})$, respectively, the FID can be defined as [3]

$FID=\left\| m-m_{\omega} \right\|_{2}^{2}+Tr\left( C+C_{\omega}-2\left( CC_{\omega} \right)^{\frac{1}{2}} \right)$ (4)

The FID was calculated using all real training images and 50,000 synthetic images generated every 10 training ticks. Lower FID scores indicate higher similarity between real and synthetic image distributions. This process ensured the high fidelity and diversity of synthetic samples used in the augmentation process.

**Signal-to-noise Ratio (SNR) Calculation**

To assess the clarity and quality of both real and synthetic images, the signal-to-noise ratio (SNR) was computed. SNR is defined as the ratio of the mean intensity of the signal to the standard deviation of the background noise [4]:

$$SNR=\frac{\mu_{signal}}{\sigma_{noise}} (5)$$

​where $\mu_{signal}$ is the mean intensity of the image, and $\sigma_{noise}$ is the standard deviation of the background noise. Higher SNR values indicate that the image contains more useful signal information relative to noise, resulting in clearer images [4].

For HGD lesions, the real dataset achieved a mean SNR of 2.4160 with a 95% confidence interval of [2.3357, 2.4963], while the synthetic dataset showed a slightly higher mean SNR of 2.5256 with an interval of [2.5047, 2.5465]. Similarly, for cancer lesions, the real dataset yielded a mean SNR of 2.2119 with a 95% confidence interval of [2.1525, 2.2713], and the synthetic dataset recorded a mean of 2.2405 with a 95% confidence interval of [2.2220, 2.2590]. These results indicate that the synthetic images maintain comparable, and in some cases improved, SNR characteristics relative to real images, supporting their reliability for model training and highlighting the benefit of GAN-based augmentation in generating diagnostically meaningful data


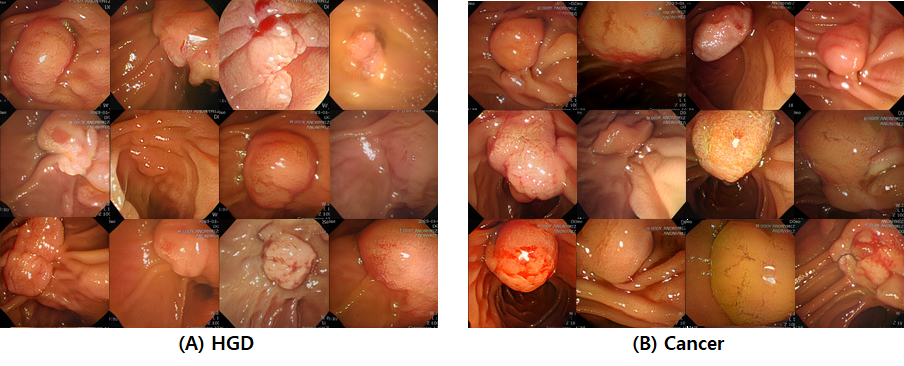


Figure S1. Representative StyleGAN2-ADA generated white-light (WL) endoscopic images of ampullary lesions.

(A) Synthetic WL images of high-grade dysplasia (HGD). (B) Synthetic WL images of cancer. Images exhibit morphologic features such as mucosal irregularity, elevation, and discoloration, faithfully reflecting real lesion characteristics used for model training.


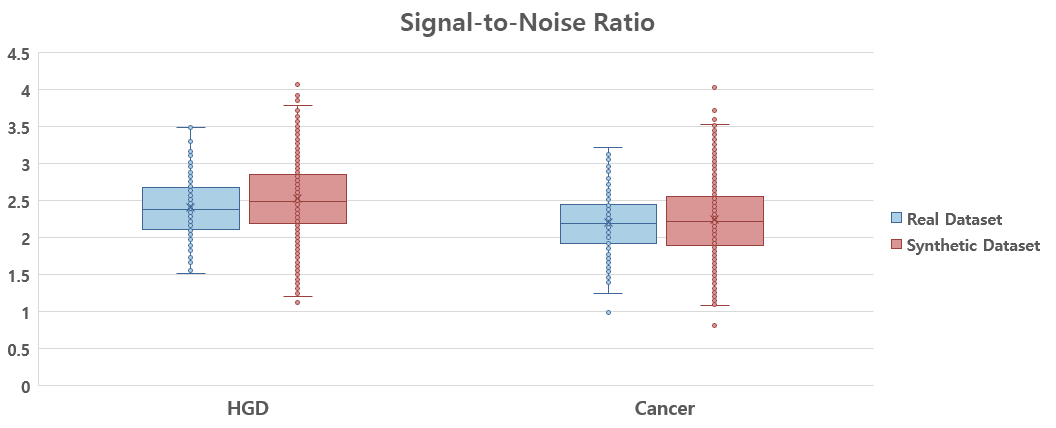


Figure S2. Comparison of signal-to-noise ratio (SNR) between real and GAN-generated images for HGD and cancer lesions

**Table S1. Number of images used in model development (number of patients)**

|  | **WL** | | | **NBI** | | |
| --- | --- | --- | --- | --- | --- | --- |
|  | **Train** | **Validation** | **Test** | **Train** | **Validation** | **Test** |
| **Normal** | 482 (104) | 122 (36) | 98 (32) | 264 (75) | 85 (27) | 65 (31) |
| **LGD** | 1,198 (123) | 415 (43) | 397 (43) | 442 (86) | 123 (30) | 98 (27) |
| **HGD** | 117 (23) | 47 (6) | 29 (7) | 49 (13) | 15 (5) | 7 (3) |
| **Cancer** | 124 (28) | 24 (9) | 23 (10) | 17 (4) | 2 (1) | 1 (1) |
| **Total** | 1,921 (278) | 608 (94) | 547 (92) | 772 (178) | 225 (63) | 171 (62) |

**Table S2. Classification performance of AoV lesion diagnosis on white-light (WL) and narrow band imaging (NBI) test sets**

|  | **WL Model** | | **NBI Model** | | **Confidence-based Voting Model** | |
| --- | --- | --- | --- | --- | --- | --- |
|  | **Accuracy** | **AUROC** | **Accuracy** | **AUROC** | **Accuracy** | **AUROC** |
| **WL Test Set** | 0.8793 | 0.8662 | 0.7057 | 0.7024 | 0.9177 | 0.9136 |
| **NBI Test Set** | 0.7544 | 0.7491 | 0.8889 | 0.8948 | 0.9356 | 0.9208 |

**Reference**

[1] M. Tan and Q. V. Le, “EfficientNet: Rethinking Model Scaling for Convolutional Neural Networks,” *in Proceedings of the 36th International Conference on Machine Learning (PMLR) (2019)*, vol. 97, 6105–14

[2] T. Karras et al., “Analyzing and Improving the Image Quality of StyleGAN,” *in Proceedings of the IEEE/CVF Conference on Computer Vision and Pattern Recognition (CVPR)* (2020), 8110–19

[3] M. Heusel et al., “GANs Trained by a Two Time-Scale Update Rule Converge to a Local Nash Equilibrium,” *in Advances in Neural Information Processing Systems (NeurIPS ’17)* (2017)

[4] M. J. Firbank, A. Coulthard, R. M. Harrison, and E. D. Williams, “A comparison of two methods for measuring the signal to noise ratio on MR images,” *Physics in medicine and biology* 44, no. 12 (1999): N261–N264. https://doi.org/10.1088/0031-9155/44/12/403
